# Lexicon of Living Medical Authors, Physicians, Surgeons, Man-Midwives, Apothecaries, and Natural Historians of All Civilized Nations

**Published:** 1844-04

**Authors:** 


### ART. XIV.

*Medicinisches Schriftsteller-Lexicon der jetz lebenden Aertzte, Wundärtzte, Geburtshelfer, Apotheker und Naturforscher aller gebildeter Völker.* Von ADOLPH CARL PETER CALLISEN, Doctor der Medicin und Chirurgie, Professor an der Königl. chirurgischen Academie zu Copenhagen, &c.—*Copenhagen*, 1830-42. Band i—xxx.

*Lexicon of Living Medical Authors, Physicians, Surgeons, Man-midwives, Apothecaries, and Natural Historians of all civilized nations.* By A. C. P. CALLISEN, M.D.—*Copenhagen*, 1803-42. Thirty Volumes.

THE above work is one of the most remarkable ever undertaken or executed by an individual. In point of labour and research, it may be compared to that of Dr. Copland, just noticed, although it emulates it in no other respect. The *Lexicon* is a mere bibliography; but it is a bibliography of a very peculiar kind, and must ever remain a monument of the astonishing industry and perseverance of the author.

As its title implies, it is devoted exclusively to the writings of contemporary medical authors, and is arranged in alphabetical order, according to the writers' names. In addition to the authors' works, it gives also, in all practical cases, a brief note of the place and time of their birth, of the place and date of their graduation, of the offices they hold, or have held, and of their actual residence, and social position. It gives the titles of the works in full, in the language in which they are composed; the date of publication, the number of editions, and the date of each, the form, size, number of pages, and frequently the price; the translations (if any) into foreign languages; and lastly, the volume and page of the journals in which the individual work has been reviewed. Another very marked feature in the Lexicon is, that in addition to the notice of distinct works published separately by any author, it gives the title of every paper printed by him in any periodical work, as well as a reference to the German journals in which such paper has been translated or noticed!

The first portion of the work, comprehending the complete alphabet of authors, closes with the twenty-first volume, published in the year 1835, only five years from the commencement of the publication. Volumes xxii and part of xxiii, contain the anonymous works that have appeared since the year 1780; and then follows the catalogue of all the journals, transactions of societies, and collections of the works of individual authors since the same date. This portion of the work occupies nearly three volumes, terminating with the twenty-fifth. The exposition of the contents of these numerous works is very minute and complete. Last of all comes the *Nachtrag* or appendix, containing emendations of, and additions to the articles in the preceding parts, a catalogue of the more recent periodical literature, and accounts of the writings of authors who have died since 1830, the date of the commencement of the undertaking. This last portion of the work is still incomplete, the last volume which has reached us, the thirtieth, containing only the letter M. It appeared at Copenhagen in 1842.

The magnitude of this work, and the astonishing labour expended in its composition, must appear sufficiently evident from the above account of it. It is stated in the preface to the twenty-fifth volume, that the first twenty-one volumes contain the names of 28,061 authors, and the titles of 68,404 works or papers written by them, without reckoning the innumerable references to new editions, extracts, reviews, &c., under each work. In the twenty-second and twenty-third volumes, there are noticed 3,783 anonymous works published since 1780; and in the twenty-fourth and twenty-fifth, 2,240 collections of authors' works, transactions of societies and journals. The contents of the appendix, which will probably extend to seven or eight volumes, will swell this list not a little.

Although we think the plan of Professor Callisen's work defective in some respects, we consider it invaluable as a mine of modern medical bibliography. In point of completeness it has no rival in any modern production; and its accuracy, as far as we are able to judge, is, all things considered, extraordinary. When it is completed with its various indexes of classified contents, &c., it will be indispensable in every medical library. It is a curious fact that an English or French physician or surgeon, desirous of obtaining a complete list of the medical writings of one of his countrymen, that have appeared during the last sixty years, has no other sure resource but to go to a work written and published in Copenhagen.

We cannot conclude this brief notice of so great a work, without offering to its most industrious and public-spirited author, the expression of our respect and gratitude. We have reason to believe that in a mercantile point of view, the Lexicon has not been merely unprofitable but the occasion of great pecuniary loss to Professor Callisen; and nothing but the noblest and purest principles of action could have led him to persevere, during so many years, in bringing it to a conclusion. We think he has thus fairly established a claim on the support of the medical profession of all countries; and it will give us much gratification if the present imperfect notice of his labours should induce the more wealthy of our brethren, and the public medical libraries, to add the Schriftsteller-Lexicon to their stock of books.

